# Treatment Options for Brachial Plexus Injuries

**DOI:** 10.1155/2014/314137

**Published:** 2014-04-14

**Authors:** Vasileios I. Sakellariou, Nikolaos K. Badilas, Nikolaos A. Stavropoulos, George Mazis, Helias K. Kotoulas, Stamatios Kyriakopoulos, Ioannis Tagkalegkas, Ioannis P. Sofianos

**Affiliations:** ^1^Department of Orthopaedic Surgery, Hospital for Special Surgery, 535 East 70th Street, New York, NY 10021, USA; ^2^Department of Orthopaedic Surgery, General Hospital of Levadia, 32100 Levadia, Greece; ^3^1st Department of Orthopedic Surgery, Athens University Medical School, Attikon University General Hospital, Chaidari, 12462 Athens, Greece

## Abstract

The incidence of brachial plexus injuries is rapidly growing due to the increasing number of high-speed motor-vehicle accidents. These are devastating injuries leading to significant functional impairment of the patients. The purpose of this review paper is to present the available options for conservative and operative treatment and discuss the correct timing of intervention. Reported outcomes of current management and future prospects are also analysed.

## 1. Introduction

The incidence of brachial plexus injuries (BPI) has rapidly increased over the last 50 years, due to technological advancements in transport, and specifically the motor vehicle field, during the 20th and 21st century. Developments in microsurgery now offer us new modalities to improve the clinical outcome of brachial plexus lesions. The last 30 years have seen good progression in brachial plexus injury outcomes. Apart from nonoperative (conservative) management, through which we can achieve reasonable mobility with the help of rehabilitation and physiotherapy, we also have new surgical options, such as neurolysis, nerve repair, use of nerve grafts and nerve transfer, and palliative surgical procedures to obtain the best functional results, such as tendon transfer or functioning free muscle transplantation and arthrodesis.

### 1.1. Timing of Surgical Procedure

The most critical point while planning a surgical procedure in brachial plexus injuries is the delay between the accident and the intervention. Indications for emergency operative procedures include vascular injury, open penetrating injuries, and open infected crushing/stretching wounds. Almost emergent surgical operation during the first or second week is recommended for complete traumatic palsy of the C5-T1 root [1]. After the 3rd month surgical operation is recommended for traumatic palsy injuries with no clinical sign of functional restoration or electromyography signs of denervation. Another group of patients recommended for surgical exploration is that with clinical and EMG signs of recovery of distal branches instead of proximal axons. Perioperative assessment of the lesion is more accurate after Wallerian degeneration has occurred. Lesions due to iatrogenic etiology should be surgically explored at an earlier stage, especially when electromyography reveals complete denervation with no signs of functional recovery.

Nerve reconstruction is not recommended for traumatic lesions more than 9 months after the accident [2–5] although there have been reports of successful procedures after 9 months [4–7]. The patient's age at the time of operation is a significant prognostic factor that should be taken into consideration with respect to the delay between the accident and the surgical intervention. Some BPI surgeons recommend that patient age above 50 years is a contradiction for surgical exploration by itself [8]. Other surgeons have reported adequate functional recovery in elderly patients [6, 7]. BPI operative procedures are generally suggested in younger age groups that have better clinical outcomes. Preoperative prioritization is rather important. The surgeon needs accurate information on the preoperative planning so as to be ready for any difficulties and variations during the procedure. Flexion of the elbow is always the first function to attempt to restore, followed by shoulder stability, abduction, external rotation, and scapula stabilization. Reconstruction of the long thoracic nerve should follow whenever possible. The motor function of the radial nerve is often recovered as shown by the recovery of the triceps rather than wrist and finger extensors. Although functional recovery of the ulnar and median nerve is not in our spectrum as research is further focused in this field, in 1986 Gu et al. reported M3 recovery of wrist and finger flexors in 5 of the 8 patients who underwent contralateral C7 transfer [9]. Median nerve sensitivity should be restored by any means as significant improvement has been noted in patients' pain even without motor recovery. Berman et al. described the use of intercostal nerves in delayed (over a year posttrauma) interventions aiming only at pain relief after BPI. Significant pain relief 8 months postoperatively was noted in 16 out of 19 patients [10]. Furthermore, Giuffre et al. and Hatoori et al. mentioned the need of restoring the protective sensation of the median nerve [11, 12].

## 2. Treatment

### 2.1. Open Wounds

Open wounds in BPI are uncommon and vary from small penetrating injuries to high energy injuries leading to amputation. In the case of acute nerve dissection it is mandatory to carry out repairs quickly given the general clinical status of the patient. Thoracic injuries and trauma of major vessels frequently follow these types of BPI.

In cases of delay between injury and intervention, scheduling of second time procedure should take place. During this period, electromyography can be used to record spontaneous potentials with and without stimulation and provide us with the appropriate evidence for preoperative planning 4–6 weeks after the injury. By this period, the nerve injury will have demarcated enabling nerve repair. In such cases nerve grafts are recommended, rather than end-to-end anastomosis and nerve reconstruction.

Open penetrating injuries like low energy civilian gunshot injuries do not have to be explored directly. Such injuries are almost always due to neuropraxia [13].

### 2.2. Closed Wounds

In the case of closed BPI wounds and when there are no other emergent injuries, surgical exploration and recovery may not take place immediately. Recommendations include evaluating the situation, managing pain, and starting rehabilitation. Electromyography may take place after 3 or 4 weeks and CT/myelography or MRI after 6–8 weeks, if denervation persists. In cases of lack of functional recovery or loss of neurological recovery surgical treatment may be considered after 3–6 months. If clinical exam suggests preganglionic lesions, confirmed by imaging results treatment strategy would likely require nerve transfer.

## 3. Conservative Treatment

The aim of conservative treatment is to maintain the range of motion of the extremity, to strengthen the remaining functional muscles, to protect the denervated dermatomes, and to manage pain.

Chronic edema may appear as a result of hypokinesia, loss of vascular tone due to sympathetic denervation, and any other soft tissue injury. Keeping the extremity raised and splitting and tensile banding may decrease edema. This should be followed by physiotherapy otherwise stiffness may be the final outcome, especially in the hand.

Management of anaesthesia-denervated dermatomes is the same as with diabetic neuropathy, with the patients avoiding extreme temperatures.

Pain management may be a rather difficult procedure. Significant pain is observed in complete palsy of the brachial plexus especially in root avulsions. Pain may be not only excruciating and exhausting for the patient but it can affect the rehabilitation procedure and as a result is of great importance. This is precisely when drugs should be used. NSAIDs and opioid drugs help us during the first stages but do not appear to help with neuropathic pain, which requires careful use of antiepileptic drugs (gabapentin and carbamazepine) or antidepressants such as amitriptyline. About 30% of patients report significant pain relief with this type of treatment. Biofeedback, punctuation, hypnosis, and percutaneous nerve stimulation have mixed results. Nashold in 1984 described the Dorsal Root Entry Zone (DREZ) operation for rather persistent pain [14, 15]. The operation is based on the effort to inhibit the nerve signal transmission from the secondary central sensory centrally, by destroying them [14, 15].

## 4. Surgical Treatment

A variety of surgical procedures have been reported to improve the functional outcome. Which one is appropriate depends on the type of lesion. The types of surgical procedures are as follows.

### 4.1. Neurolysis

When the nerve lesion is in continuity, neurolysis may help. It is of great importance to maintain the interfascicular structure and the nerve sheath. Because of the risk of vascular damage we prefer not to conduct interfascicular neurolysis; instead an anterior epineurectomy is performed, excising the fibrous tissue. Use of direct nerve stimulation before and after neurolysis helps us demonstrate the improvement in nerve conductance. The clinical outcome of neurolysis is not easy to identify as any functional improvement may be the result of many factors other than neurolysis itself.

### 4.2. Nerve Grafting

Nerve grafting is the predominant technique for clear cut injuries with a healthy proximal stump and with no axial damage. The outcome is influenced by the length of the nerve graft, the presence of scar tissue at the wound site, the number of grafts used, the presence of a healthy proximal stump available for grafting and the nerve gap to be covered. Postoperatively, the nerve should respond to somatosensory evoked potentials (SEPs) and the conductivity of the stimulated spinal roots should be verified [14–19], while controversies remain on the evaluation of C5 through SSEPs. This procedure is the basis of current surgical treatment of postganglionic spinal nerve injury. When damage is extensive, prioritization of certain nerves for repair by grafting is necessary, especially those associated with elbow flexion, shoulder abduction and sensation of the forearm.

The sural nerve, the sensory branch of ulnar nerve, and the medial cutaneous nerve of the forearm are the usual donor nerves. The sural nerve may provide up to 40 cm of neural tube. The donor site should be in situ until the recipient site is ready. Immediately before the grafting procedure the donor nerve should be inverted so as to minimize any loss of axial branches. Generally, use of nerve grafts shorter than 10 cm results in better functional and clinical outcomes compared with longer grafts [1, 5, 20, 21].

The use of free nerve grafting for peripheral functional recovery seems to provide poor results compared to the reconstruction of more proximal lesions [22]. Another choice is vascular nerve grafts when the ulnar nerve is often used [23]. In such cases, the ulnar nerve is divided into smaller grafts, the size of the sural nerve, so as to increase the chance of success [24]. However, vascular nerve grafts do not seem to outclass free nerve grafts with respect to recovery and functional improvement [25]. Surgical technique is a significant factor for the outcome of nerve grafting. Our aim is always to achieve the best fixation without any tension at the point of anastomosis of the graft [5].

### 4.3. Neurotization

This type of procedure is used for preganglionic root injury in BPI. Neurofibres are transferred to an irreparable paralytic. Motor branches are used as donors aiming to achieve motor reinnervation, respectively, to the sensory ones. The nerve transfer may be extraplexus or intraplexus. Intraplexus transfer options include intact nerve roots. Other choices include the use of the medial thoracic nerve and inferior medial cord/ulnar nerve. Oberlin et al. described nerve transfer to the biceps muscle using part of the ulnar nerve for C5-C6 avulsion of the brachial plexus [26, 27].

Extraplexus transfer options include the use of intercostal and spinal accessory nerves. The phrenic nerve—accessed using an anterior neck approach—and deep motor branches of the cervical plexus (C3-C4) may be used as donor nerves. Apart from the use of deep motor branches of the cervical plexus the rest of the donor nerves may restore elbow flexion and result in M3 biceps strength in approximately 75% of patients [4, 27]. The Oberlin technique is recommended for patients with avulsion of the upper roots and intake of the lower roots of the brachial plexus [4, 26]. Nerve transfer to biceps muscle using part of the ulnar nerve in upper arm type brachial plexus injury provides good functional and clinical outcomes [26, 28, 29]. The use of Oberlin's technique led to M3 or more biceps strength in 94–100% of patients and M4 biceps strength in 75–94%. However, the procedure requires intake of lower roots of plexus. The spinal accessory nerve is another option [30]. The accessory nerve is a pure motor nerve but only one or two of its final branches should be used so as to maintain the normal function of the trapezoid. No deficit related to the transfer of intercostal nerves has ever been noted, but these nerves may be easily damaged in patients with pneumothorax, multirib fragments, or spinal cord trauma. The phrenic nerve is a good donor nerve but we should not forget its contribution in respiratory function and the possible dangers, especially in patients with simultaneous intercostal nerve transfer. Gu et al. revealed no significant reduction in respiration following phrenic nerve transfer for brachial plexus motor neurotization.

Moreover, the use of medial pectoral nerve (MPN) to axillary nerve transfer is a valid choice of treatment to restore shoulder stability [31].

Transfer of the C3-C4 cervical root may affect the stability of the scapula. Transfer of the hemicontralateral root of C7 is another good option for brachial plexus injuries with total avulsions [9, 32–34]. The clinical indication is total traumatic damage of the brachial plexus with multiple avulsions and limited nerves available for transfer. The contralateral C7 root may be enlarged with the use of a vascular ulnar nerve graft in patients with C8-T1 avulsion injuries and the most commonly used is the median nerve [9]. Neurological deficits have been reported after such a procedure, even in C7 sensory function [9, 32–35]. Another option is to transfer the nerve of the long head of the triceps to the axillary nerve so as to reinnervate the motor function of the deltoid muscle [36]. As was described by Witoonchart et al. and Leechavengvongs et al. it is a promising procedure in combination with the transfer of the accessory to the suprascapular nerve [37, 38].  Table 1 shows the usual donor and recipient nerves. A rather significant factor that should be taken into consideration preoperatively is the total axial number of every potential donor nerve. The total axial number depends on the donor nerve itself, as shown in Table 2.

The musculocutaneous nerve has 6000 motor branches while the anterior branch of the axillary nerve has 2700 branches [37].

Some surgeons prefer to use intraplexus nerve transfer due to better outcome in patients with devastating paralysis [39]. However some extraplexus nerve donor sites also provide good clinical results. As an example the use of intercostal nerves for functional reconstruction of the elbow or shoulder provides 70% up to over 90% of best results.

The end-to-side neurorrhaphy with removal of the epineurial sheath is a rather up to date surgical technique [40]. Using this technique we managed to transfer the stump of a denervated, paralytic nerve to a new healthy one without losing its functionality.

A wide range of prognostic factors may affect the outcome of any BPI surgical procedure. This is why it is not safe to predict any clinical outcome. The literature reveals better clinical outcomes for younger patients and upper trunk injuries. This may result from the shorter nerve gap that has to be bridged. Functional restoration of the hand due to lower trunk BPI remains the most challenging part of microsurgery.

### 4.4. Secondary Operations

In the absence of spontaneous recovery or when the first surgical procedure does not provide satisfactory outcomes then a second operation may be required. In such cases there should be specific signs of neurological denervation or no possibility of neurological recovery, or sufficient time should have passed with no functional improvement. Arthrodesis, tendon transfer, and functional free muscle transplantation are our favored treatment options.

### 4.5. Arthrodesis

In complete brachial plexus traumatic injuries, arthrodesis resulting in shoulder stabilization gives the surgeon the opportunity to collect all potential nerve grafts so as to proceed with any available procedure. On the other hand, in upper level BPI with rather unstable and painful shoulders, arthrodesis could be a definite solution. When planning shoulder arthrodesis certain parameters should be taken into consideration. Firstly, good thoracic-shoulder functionality is of great importance. Secondly, the motion mobility of the peripheral hand is important as shoulder arthrodesis has no clinical effect on a paralytic hand whatsoever. The acromioclaviclural joint, sternum-claviclural joint, and spaculothoracic joint should be intact. Any dysfunction may affect the success of arthrodesis.

The shoulder should be fused with only 20 degrees of abduction, 30 degrees flexion, and 30 degrees of internal rotation to allow the patient to be independent in his daily life with a mean range of 60 degrees abduction and flexion through the scapulothoracic joint [41].

### 4.6. Tendon Transfer

Tendon transfers are useful in restoring upper extremity function after BPI. Basic principles must be followed if transfers are to be successful. An absolute indication for tendon transfer is upper or lower brachial plexus traumatic injury with only partial paralysis. Using the latissimus dorsi muscle, which is used for elbow flexion rather than elbow extension and finger flexion, provides nonsatisfactory results (in the muscle grading system of Seddon: M3 or even less muscle strength). This is due to the fact that the innervation of the latissimus dorsi stems from the C6, C7, and C8 roots. After tendon transfer, muscle strength is not restored to preinjury levels, in most cases with the loss of at least one grade on the measurement of muscle strength.

Many tendon transfer techniques have been described for treating partial shoulder paralysis. A decision should be taken only when all options have been assessed. Among the most common procedures are the following:

- trapezius to deltoid transfer as described by Elhassan et al. in 2000 to restore abduction of the shoulder [42];
- latissimus dorsi transfer as described by L' Episcopo, to improve external rotation. The L' Episcopo technique may be used at the same time as removing part of the anterior joint capsule and releasing the subscapular and pectoralis major muscle or even with humerus external rotation osteotomy [36, 42–44];
- Anterior transfer of the posterior branch of the deltoid muscle to restore nonfunctional anterior segment.

Restoration of elbow flexion is of great importance for a good clinical and functional outcome. Depending on the level of injury and the degree of reinnervation there are different types of surgical procedure. The surgical goal is to restore good muscle strength through a range of elbow motion (30 to 130 degrees). The most commonly used procedures are as follows:

- transfer of the common origin of the flexor forearm muscles to a proximal section as described by Steindler (1918) [45]. Better results can be achieved via transfer of the common origin of the flexor forearm, 5 cm closer to the medial epicondyle, with bone attachment being preferred to periosteal. This type of procedure provides nonsatisfactory outcomes when used in cases of complete elbow paralysis. In the case of resting elbow flexion following nerve transfer or as an accessory to other tendon transfer techniques, it provides us with better clinical results [46]. The Steindler technique may lead to disappointing outcomes such as elbow stiffness or over pronation;
- transfer of latissimus dorsi muscle to the tendon of the biceps brachialis provides great muscle strength, but this muscle is often denervated;
- transfer of pectoralis major brachial branch tendon to brachial biceps (Clark technique). A fused shoulder is required for the best postoperative result;
- transfer of triceps tendon to biceps provides good results not only with respect to muscle strength but also aesthetically [47].

As mentioned earlier, restoration of good clinical functionality is perhaps the most difficult goal for an orthopaedic surgeon. The first factor to take into consideration is that we are attempting to achieve a flexible joint with a good range of motion. Therefore, tendon transfer on a stiff joint is pointless. In the case of a stiff joint, intensive physiotherapy is required to achieve an acceptable range of motion even with surgical release. In case of complete sensor defect, we do have to restore it due to the fact that a hand with no tactile sensation is very dysfunctional limp.  Table 3 shows hand functions and the tendons that are mostly responsible for these functions.

### 4.7. Functioning Free Muscle Transplantation

Functioning free muscle transplantation (FFMT) is the transfer of a muscle using microvascular anastomoses for revascularization and subsequent microneural coaptation to the recipient motor nerve for reinnervation.

Of the surgical techniques that have been described in this paper both nerve grafting and nerve transfer may provide sufficient muscular reinnervation. It should be noted that these procedures provide better results when conducted within the first 6 to 9 months [6, 27, 30, 48–53], even though some researchers have reported that muscular strength may be restored within the first 12 months postoperatively [54, 55].

The changes stemming from muscle denervation can be biochemical and/or morphological. Disorganization is complete after 2 years of denervation and muscle is eventually replaced by fat tissue. Within 2-3 months of the posttraumatic period denervated muscle fiber loses 50% of its diameter due to atrophy. In many cases, a delay in surgery or complete avulsion of the brachial plexus limits our ability to achieve a good outcome. Apart from the references for compensatory functional outcome of the range of motion of shoulder and of elbow with the use of the nerve transfer and nerve grafting techniques, always within the appropriate period, functional restoration of the hand is often disappointing. For this reason, functioning free muscle transplantation in addition to nerve transfer and grafting should be considered in cases delayed more than 9–12 months posttraumatically [28, 56–59]. Restoration of elbow flexion and wrist extension in brachial plexus paralyses and even complete brachial plexus avulsions may succeed using the reinnervated free-muscle transfer technique [57, 59–62].

As in the case of tendon transfer, functioning free muscles should have sufficient strength and range of transfer compared to the denervated muscle they are replacing [63]. Moreover, the candidate donor muscle should have adequate vascularity and be innervated by appropriate nerves so as to allow reinnervation through direct nerve transfer. The shortest distance between the donor nerve and the transferred muscle provides the shortest reinnervation time. In addition, the donor muscle should not have an essential function, in order to avoid significant impairment of hand function at the affected sites. Studies suggest that the rectus femoral and gracilis muscles are the most common donor muscles [56, 57, 59–62, 64–67] followed by the latissimus dorsi, major pectoralis muscle, and tensor iliotibial band muscle [68–72].

Free functioning muscle transfer is recommended for two purposes in BPI: (1) to restore elbow flexion in patients after delayed presentation and in the absence of alternative solutions; (2) in patients who present within the first 6 months after trauma in combination with nerve grafting and/or nerve transfer. A simple or double free muscle transfer technique is used. By using simple free functioning muscle transfer we can achieve compensatory elbow flexion in cases of delayed treatment in which nerve grafting and transfer to reinnervate the brachial biceps are insufficient [58, 59, 63, 64, 72–79].

**Figure 1:**
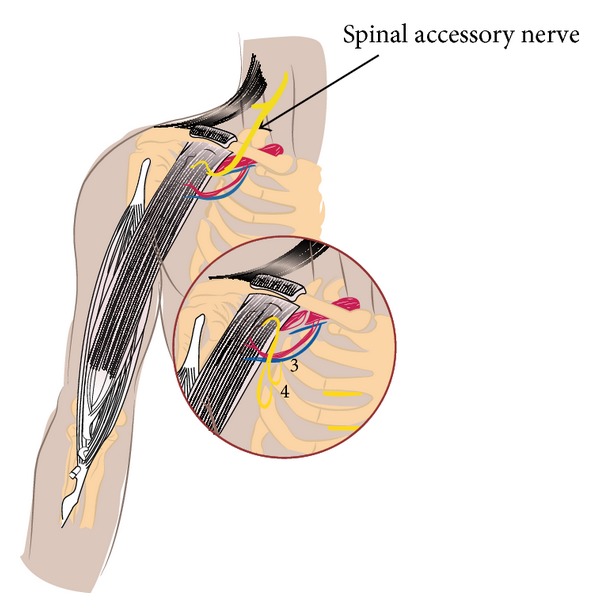
Simple functioning free gracilis muscle transplantation to restore elbow flexion. The gracilis is attached proximally to the clavicle and distal to the biceps brachialis tendon. Range of motion is provided through innervation from spinal accessory nerve or from the 3rd and 4th intercostal nerve. Vascularization is provided from the thoracoacromial vascular branches.

In some cases free functioning muscle transfer may be useful for restoring shoulder abduction and elbow and finger flexion [57]. The following figure illustrates the technique of gracilis muscle transfer (Figure 1).

**Figure 2:**
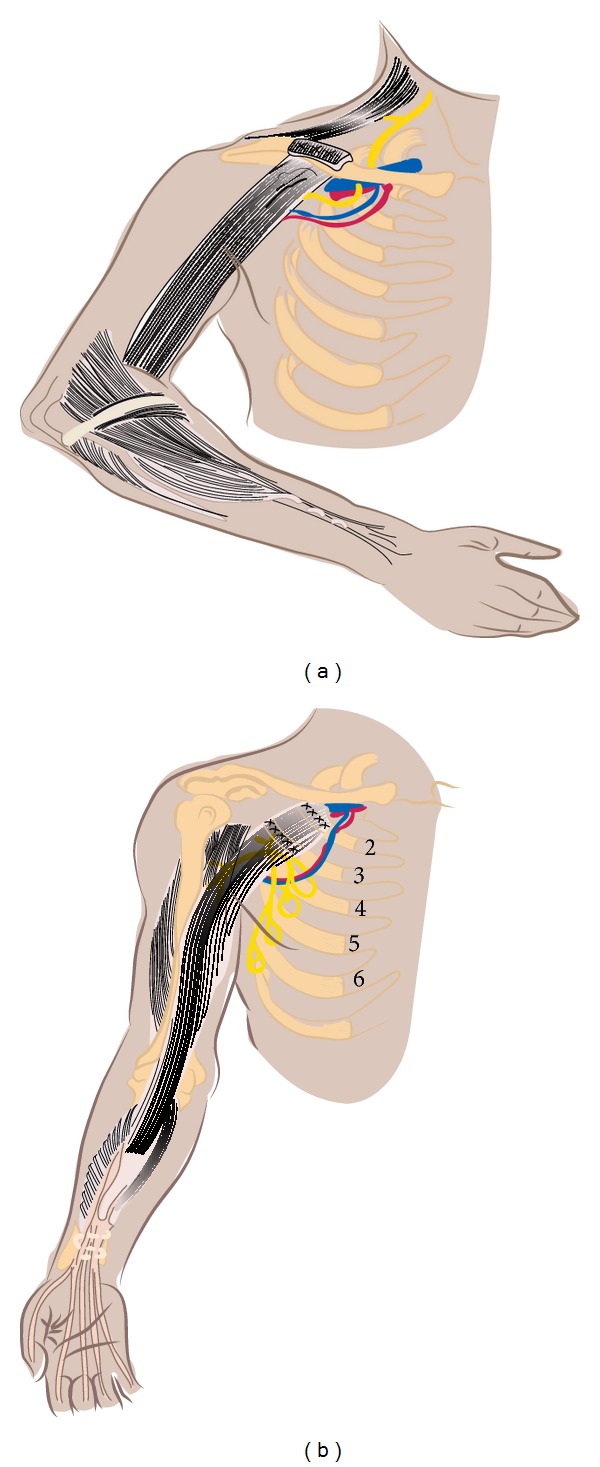
Schematic presentation of Doi's technique. (a) Transfer of the first gracilis muscle and fixation to the clavicle provide adequate elbow flexion and finger extension. (b) Transfer of the second gracilis and fixation to the second rib provide finger flexion.

Doi et al. described a technique that may provide shoulder stabilization and functional outcome in addition to active flexion and extension of the elbow, sensitivity, primary grip, and pinch strength in patients with four or five levels of avulsion [59–62, 79–81]. Restoration of sensory function is imperative when prehensile function is restored after irreparable brachial plexus injury. Moreover, the double free muscle technique uses the length of the gracilis, which allows us to mobilize a rather distant arthrosis, and the shortest distance between the muscle and the donor nerve, which provides the shortest reinnervation time. Nerve reinnervation of the gracilis is provided by the spinal accessory nerve immediately after transfer of the muscle to the clavicle. In this way, elbow flexion and wrist or finger extension may be restored. Transfer of the second gracilis muscle involves transferring the muscle to the second rib, and following nerve transfer from the intercostal nerves we can restore finger extension (Figures 2(a) and 2(b)). By using further intercostal nerves to restore range of motion to the brachial triceps and nerve transfer to the hand with the aim of achieving sensory recovery we may have a satisfactory outcome in terms of flexion/extension of the elbow and grip/laxity of the hand. Using this surgical technique Doi managed to achieve excellent elbow flexion. Moreover, by using the second gracilis he achieved more than 30 degrees active finger motion in 65% of patients [80].

### 4.8. Future Prospects

#### 4.8.1. Reimplantation of Brachial Plexus—The Role of Neurotrophic Factors

Since the 1980s many scientists have achieved axonal regeneration in central nervous system transplants through the use of peripheral nerve autografts [82–87].

Cellular death of motor neurons of the anterior spinal cord horn occurs 6 weeks following complete avulsion of the brachial plexus and only 40% of them remains at final follow-up. Direct reimplantation or the use of peripheral nerve autografts may increase the total number of remaining motor neurons by up to 80% after 6 weeks. This procedure is associated with spinal axon regeneration and functional restoration. Schwann cells are mainly responsible for the regeneration. Studies have shown that Schwann cells do not slough after dissection of peripheral axons but continue releasing neurotrophic factors and producing extracellular constituents such as laminine.

Although the fact that supraclavicular subcutaneous lesions of the brachial plexus are not associated with skeletal injuries was first described in 1911 [88] and the first attempt at reimplantation of an avulsed cervical root took place in 1979 [89], it was only in 1995 that Carlstedt et al. reported the first successful spinal cord implantation of an avulsed spinal nerve root [90]. Five years later Carlsted published the results of a number of spinal nerve root repairs and reimplantations of avulsed ventral roots into the spinal cord after brachial plexus injury [91]. He described the attempt to restore traumatic brachial plexus lesions in ten patients who were operated on within 10 days to 9 months after trauma. In all cases the sural nerve was implanted into the materia alba of the transverse horn of the spinal cord to a depth of 1-2 mm. The first signs of regeneration were noted approximately 9 to 12 months postoperatively. Muscle activity/retraction was observed in eight patients within the first year. In five of them, this type of activity/retraction remained ineffective, while in the remaining three, who were operated on relatively quickly (10–28 days after trauma), muscle power of at least Medical Research Council Grade 3 to 5 occurred within 3 to 5 years post-operatively. The authors emphasized the importance of a short time lag between the accident and surgery, recognizing it as a significant factor for a successful outcome.

Many questions remain about this type of procedure. It also has limitations. In particular, the delay between injury and the surgical procedure plays a critical role in neurological restoration. The very early results are rather promising so that future action should take place.

Moreover the surgical approach to the spinal cord was not the appropriate one as only the transverse horn was reached instead of the anterior one. It is possible that this type of approach in combination with the use of neurotrophic factors may provide better outcomes. Schwann cells of peripheral axonal grafts may provide the appropriate amount of neurotrophic factors to use in situ. Systematic use is not possible as they are rather unstable biochemically, with a short half-life, they are not able to penetrate the blood-brain barrier, and they may have side effects as their receptors are found in other tissues too. Researchers from Mayo Clinic have conducted a study on the use of biodegradable polymer grafts for the surgical repair of injured spinal cord [92]. The use of a biodegradable polymer implant has the dual advantages of providing a structural scaffold for axon growth and a conduit for sustained-release delivery of therapeutic agents. As a scaffold, the microarchitecture of the implant can be engineered for optimal axon growth and transplantation of permissive cell types. As a conduit for the delivery of therapeutic agents that may promote axon regeneration, the biodegradable polymer offers an elegant solution to the problems of local delivery and controlled release over time. Thus, a biodegradable polymer graft would theoretically provide an optimal structural, cellular, and molecular framework for the regrowth of axons across a spinal cord lesion and, ultimately, neurological recovery [92]. The question remains as to whether 3 mm and 5 mm diameter grafts may be responsible for further injury to the spinal cord. Further research should focus on the bioengineering, characterization, and experimental application of this type of implant.

## 5. Conclusion

Unfortunately the incidence of traumatic brachial plexus injuries is increasing, leading to severe problems concerning quality of life in affected patients. As they often occur in young people the social/financial consequences may be severe. Conservative treatment may help pain management and maintain some functionality or motion. Scientific and technical advances within recent years have significantly increased the importance of direct surgical operations such as neurolysis, nerve grafting, and nerve transfer, which, in combination with arthrodesis, tendon transfer, or functioning free muscle transplantation, may improve any muscle functionality to at least some degree.

However despite improvement in surgical techniques, even when these lead to an improvement in final outcome, the functionality of the upper limb is often disappointing. An even bigger problem after total avulsion injuries is the deficit in functional restoration of hand muscles, especially the lumbrical muscles. The complexity of this type of lesion requires experienced and specialized medical and paramedic personnel in order to achieve the best result for the patient. It is of great importance for the patient to know exactly the extent of injury, what he can expect from a surgical operation, and whether he may be able to participate actively in any type of rehabilitation. Factors that will influence the final result are the delay between the time of injury and surgical intervention, concomitant vascular injuries, the age of the patient, and the length of the nerve graft. A substandard type of grip followed by elbow flexion and a stable shoulder leads to a high level of satisfaction with regard to the final result.

Over the last three decades the operative techniques for brachial plexus injuries have steadily improved. However, the rate of progress now seems to have slowed down. New hopes have arisen through modern scientific studies and research on the pathophysiology of nervous tissue, pharmaceutical research on chemical factors that facilitate nerve growth, and biological research on synthetic nerve grafts as well as on the repair of avulsed nerve roots in the spinal cord marrow.

## Figures and Tables

**Table 1:** Donor and recipient nerves.

Donor nerves	Recipient nerves
Spinal accessory nerve	Suprascapular nerve or
	musculocutaneous nerve
Phrenic nerve or intact C5 root	Axillary nerve
Intercostal nerve	Musculocutaneous, long thoracic nerve, radial, and medial nerve
Contralateral root C7	Medial nerve
Nerve for the long head of biceps	Anterior branch of axillary nerve

**Table 2:** Donor nerves and number of axons.

Nerves	Number of axons
Branch to pectoralis major muscle	400–600
Phrenic nerve	800
Intercostal nerve	1300
Long thoracic nerve	1600
Spinal accessory nerve	1700
Motor branch of middle trunk	3400–4000
C7 root	16000–40000
Nerve for the long head of brachial biceps	1200

**Table 3:** Basic actions of hand muscles and the most commonly transferred tendons for their restoration.

ACTION	Tendon
Intrinsic balance	Flexor digitorum superficialis tendon
Thump oppose	Extensor indicis
	Flexor digitorum superficialis tendon
	Abductor digiti minimi
Thump flexion	Pronator teres
	Brachioradialis
	Flexor digitorum superficialis tendon
Thump extension	Brachioradialis
	Extensor indicis
	Palmaris longus
Finger flexion	Brachioradialis
	Extensor carpi radialis longus
Finger extension	Brachioradialis
	Flexor carpi ulnaris
	Flexor carpi radialis
	Extensor indicis
Wrist extension	Brachioradialis
	Pronator teres
Wrist flexion	Rare restoration
